# Gut microbiota dysbiosis in inflammatory bowel disease: interaction with intestinal barriers and microbiota-targeted treatment options

**DOI:** 10.3389/fcimb.2025.1608025

**Published:** 2025-06-27

**Authors:** Hongjun Xie, Siyan Yu, Mingyu Tang, Yating Xun, Qin Shen, Gaojue Wu

**Affiliations:** ^1^ Department of Gastroenterology, Wuxi No. 2 People’s Hospital, Affiliated Wuxi Clinical College of Nantong University, Wuxi, Jiangsu, China; ^2^ School of Medicine, Nantong University, Nantong, Jiangsu, China; ^3^ Wuxi School of Medicine, Jiangnan University, Wuxi, Jiangsu, China

**Keywords:** inflammatory bowel disease, gut microbiota, intestinal barrier, fecal microbiota transplantation, antibiotics, probiotics

## Abstract

Recent studies have deepened our understanding on gut microbiota alterations and the interaction with intestinal barrier impairments, which play a crucial role in the etiology and pathophysiology of Inflammatory bowel disease (IBD). The intestinal microbiota dysbiosis in IBD including the altered microbiota composition, decreased beneficial species and increased harmful species. The disturbed gut microbiota results in the aggravation of intestinal barrier dysfunction through regulation of antimicrobial substances in mucus layer, tight junction protein in mechanical layer and inflammatory response in immune layer. The therapeutic options targeted on the microbiota including antibiotics, probiotics and fecal microbiota transplantation (FMT) exhibit efficacies and limitations in the treatment of IBD. Reasonable single or combined use of these treatments can restore intestinal microecological homeostasis, which further contributes to the treatment of IBD. This review analyzes the underlying mechanisms for the interaction between microbiota alterations and gut barrier dysfunction in IBD; meanwhile, it provides new insights into the microbiota-targeted therapeutic options IBD, including the benefits, risks and limitations of antibiotic and probiotic therapies, unresolved clinical application strategies for FMT, and combination administrations of antibiotics and FMT.

## 1 Introduction

Inflammatory bowel disease (IBD), a complicated group of diseases mainly including Crohn’s disease (CD) and ulcerative colitis (UC), is characterized by the chronic mucosal or transmural inflammation of the gastrointestinal tract with notable impairment of intestinal barrier (Ungaro et al., 2017; Dolinger et al., 2024). The main symptoms of IBD, including diarrhea, hematochezia, abdominal pain, fever and malnutrition (Steinwurz et al., 2023), seriously reduce the quality of life and work ability. Due to the early disease onset and high incidence in recent years, IBD is predicted to present a high burden with continued growth until 2050 (Wang et al., 2024).

It has been demonstrated that gut dysbiosis, mainly referring to the altered composition, decreased diversity and shifted functional capacities of gut microbiota and proliferation of pathogens, are highly associated with the etiology and pathology of IBD (Schaubeck et al., 2016; Weingarden and Vaughn, 2017; Lopetuso et al., 2023). Compared to the healthy population, the diversity and stability of the gut microbiota in IBD patients are significantly reduced (Hu et al., 2022). The gut microbiota dysbiosis, exhibiting an interaction with the intestinal barrier dysfunction, contributes to the intestinal inflammation in IBD. The gut microbiota-targeted therapy in IBD, including antibiotics, probiotics and fecal microbiota transplantation (FMT), seems to show divergent outcomes on gut microbiota. Antibiotics, commonly used to treat bacterial infections, can lead to a decreased abundance and an altered composition of intestinal microbiota. Contrarily, treatments of probiotics and FMT, respectively supplementing exogenous beneficial bacteria and transferring fecal microbiota from healthy donors to the digestive tract of recipients, restore the intestinal microecology (Lopetuso et al., 2023).

This review aims to explore: 1) gut microbiota dysbiosis-related pathology in IBD, focusing on the interaction between gut microbiota dysbiosis and intestinal barrier impairment; 2) therapeutic strategies targeting gut microbiota in IBD, focusing on the indications, benefits and risks of treatment of antibiotics, probiotics, FMT, and their combination.

## 2 The interaction of intestinal microbiota dysbiosis and intestinal barriers in IBD

The human digestive tract contains a vast array of gut microbiota, exhibiting interactions with intestinal barrier, which are essential for multiple physiological functions including colonization resistance to pathogenic infection, regulations of the metabolites and modulations of the mucosa immune response (Morrison and Preston, 2016; Rowland et al., 2018).

**Figure 1:**
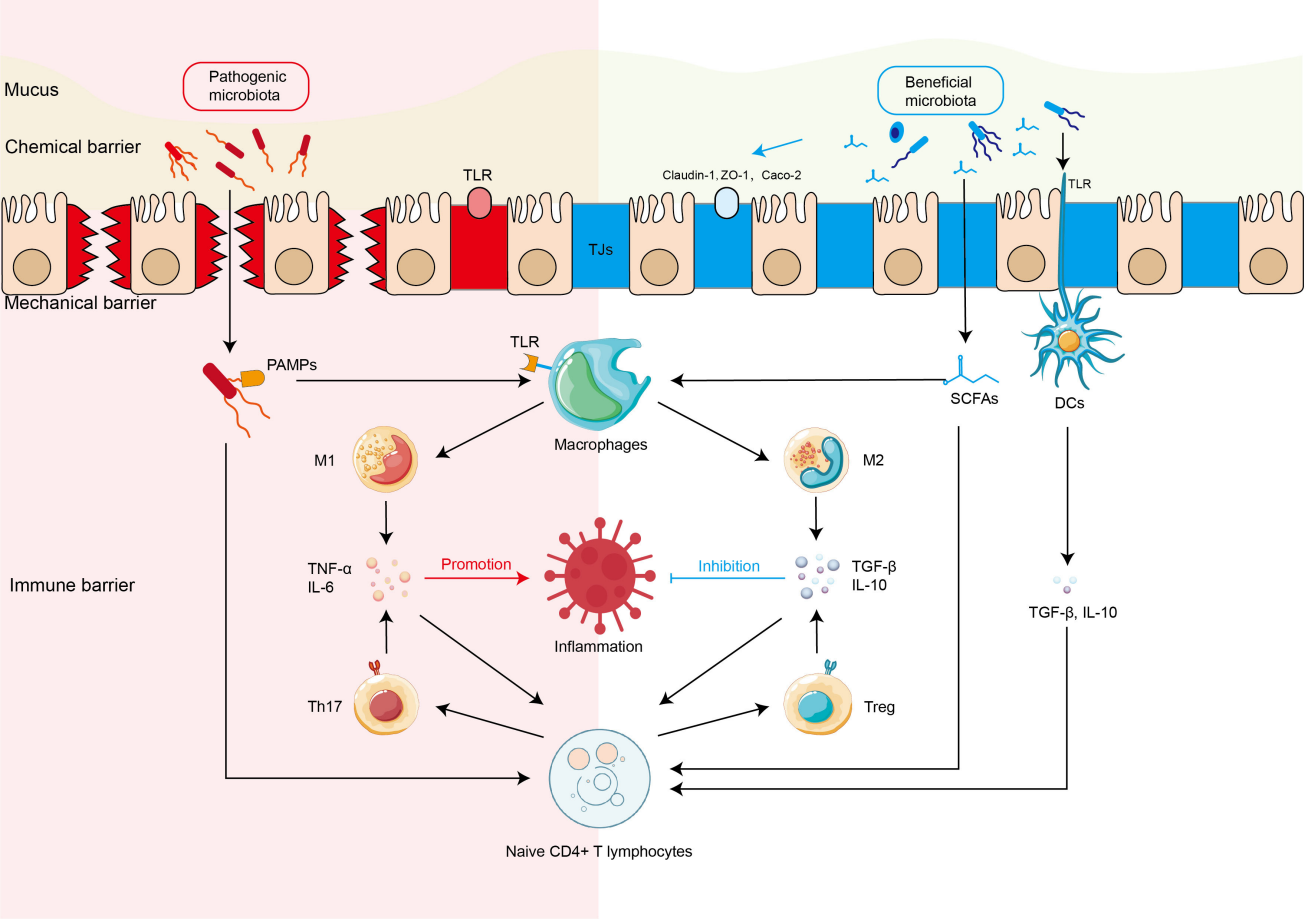
Interaction between intestinal microbiota and intestinal barriers in the IBD Harmful and beneficial bacteria regulate the expression of tight junction proteins of mechanical barrier and activation of intestinal immune cells (immune barrier) including macrophages, Th17 cells, dendritic cells (DCs) directly or indirectly through microbiota-derived short chain fat acids (SCFAs), which further regulate the production of inflammatory or anti-inflammatory cytokines in the pathophysiology of inflammatory bowel disease (IBD). DCs, dendritic cells; M1, M1 macrophage; M2, M1 macrophage; SCFAs, short-chain fatty acids; Tregs, regulatory T cells; TJs, tight junctions.

It is still controversial whether the state of microecological disorder is the one of the triggers or secondary manifestation of IBD. However, as evidenced by multiple studies (Qiu et al., 2022; Yadegar et al., 2024), the gut microbiota of IBD patients is characterized by a notable reduction in diversity and a profound alteration in bacterial construction (Nishida et al., 2018; Fu et al., 2024), including proliferation of pathogenic or other harmful species, and decreased abundance of beneficial species. Furthermore, The interaction between the gut microbiota dysbiosis and the impaired intestinal barrier, including dysfunction of chemical barrier, mechanical barrier, and immune barrier (Qiu et al., 2022), is highly involved in pathophysiology of IBD (Yue et al., 2019) (**Figure 1**).

### 2.1 Alteration of gut microbiota in IBD

The stable community of gut microbiota, also known as the intestinal microbial barrier, plays multiple physiological roles, such as preventing the colonization and proliferation of harmful microorganisms, producing beneficial metabolites and vitamins, degrading nutrients, and regulating the intestinal immune and inflammatory reactions (Sugihara and Kamada, 2024). The gut microbiota, engaging in competition with pathogens for nutrients such as amino acids, sugars, metals and respiratory electron acceptors, exerts an effect of colonization resistance to pathogenic microorganisms (Stecher, 2021). For instance, commensal Escherichia coli (E. coli) competes with pathogenic E. coli for carbohydrates, amino acids, organic acids, and other nutrients (Kamada et al., 2012). Similarly, Phascolarctobacterium competitively inhibits Clostridioides difficile (C. difficile) by reducing the availability of luminal succinate, a key metabolite necessary for the growth of C. difficile (Nagao-Kitamoto et al., 2020).

The diversity of both the mucosa and fecal microbiota reveals significant reductions in IBD. Investigation of microbiomes via a metagenomic sequencing revealed that mucosal microbial genes of IBD patients reduced by 25% in comparison with those of healthy controls (Qin et al., 2010). The fecal microbiota analyses, mainly representing those of resident luminal bacteria, have exhibited the altered composition and abundance of gut flora involved in the microbiota dysbiosis in IBD. At the phylum level, the predominant gut bacteria are Firmicutes and Bacteroidetes, which comprise 80–90% of the population, and secondary ones include Actinobacteria, Proteobacteria, and Fusobacteria (Clemente et al., 2012). However, IBD patients have been reported to show a marked decrease in the abundance of Firmicutes and an increased abundance of Proteobacteria (Nishino et al., 2018; Yang et al., 2021). The alteration of Bacteroidetes in IBD patients remains controversial (Walker et al., 2011; Xu et al., 2022; Fu et al., 2024), which may be attributed to variations in samples, and activities of IBD (Matsuoka and Kanai, 2015).

At the genus level, IBD patients exhibit a reduced abundance of beneficial bacteria, including Faecalibacterium and Roseburia (Morgan et al., 2012; He et al., 2019), which are known as key producers of short-chain fatty acids (SCFAs) (Rossi et al., 2016; Lloyd-Price et al., 2019) with the anti-inflammatory properties (Machiels et al., 2014; Nie et al., 2021) It has also been indicated a notable decrease in mucosa-associated Bifidobacterium in UC patients (Mylonaki et al., 2005) as well as a significant reduction in some special species of Bifidobacterium in CD patients, including Bifidobacterium bifidum (B. bifidum), Bifidobacterium longum, Bifidobacterium adolescentis, and Bifidobacterium dentium (Gevers et al., 2014). IBD patients show lower abundance of Lactobacillus, which exhibits weaker adhesion to epithelial cells (Najafi et al., 2022). Supplementation with these reduced beneficial bacteria can be used as a treatment option for IBD, as detailed in the Probiotic treatment section (Section 3.2). Examples of the well-recognized probiotic treatments are as follows: Bifidobacterium and Lactobacillus are administrated as traditional probiotics to provide therapeutic benefits in the treatment of IBD (Jakubczyk et al., 2020; Roy and Dhaneshwar, 2023). In addition, treating Dextran Sulfate Sodium (DSS)-induced UC in mice model with Akkermansia muciniphila (Akk), another species of gut bacteria reduced in IBD (Png et al., 2010), could alleviate the colonic inflammation (Bian et al., 2019).

The abundance of pro-inflammatory bacteria is increased in IBD patients (Tsai et al., 2025). Several pathogenic microorganisms are associated with the aggravation or progression of IBD, such as C.difficile (Bosca-Watts et al., 2015), E. Coli (Rahman et al., 2014), Mycobacterium avium (M.avium) (Feller et al., 2007), Campylobacter and Salmonella enterica (Gradel et al., 2009). Additionally, Ruminococcus gnavus (R.gnavus) is a resident bacterium in healthy individuals, whereas is particularly enriched in CD patients. It has been reported to induce the dendritic cells (DCs) to produce pro-inflammatory cytokines in CD (Joossens et al., 2011; Crost et al., 2023).

### 2.2 Gut microbiota alteration and chemical barrier impairment

The intestinal chemical barrier is mainly composed of mucus layer, including various chemical substances, such as gastric acid, bile, digestive enzymes, lysozyme and mucins (MUCs) produced by cells of host’s GI tract, and antimicrobial substances produced by the gut microbiota (Ren et al., 2019). The outer mucus layer serves as a nurturing habitat for the gut microbiota; meanwhile, the inner mucus layer acts as a shield, keeping microorganisms away from the intestinal epithelial cells (IECs). In addition to the competition with pathogens, several beneficial bacteria produce small antimicrobial molecules called bacteriocins, which can eliminate specific pathogenic microorganisms (Dobson et al., 2012). The bacteriocins produced by lactobacilli and/or bifidobacteria, including H_2_O_2_, acetic and lactic acids and biosurfactants, show benefits in inhibiting the overgrowth of Gram-positive bacteria and pathogenic microorganisms by disrupting the cell membrane and interfering with enzyme activity (Servin, 2004). However, the reduced abundance of lactobacilli and bifidobacteria has been widely reported (Mylonaki et al., 2005; Gevers et al., 2014; Najafi et al., 2022), which might further result in the dysfunction of chemical barrier.

### 2.3 Gut microbiota alteration and mechanical barrier impairment

The intestinal mechanical barrier is mainly based on the integrity of the IECs and the tight junctions (TJs) between IECs (Goto, 2013; Kurashima and Kiyono, 2017). It contributes significantly to defending against pathogens.

The impairments of intestinal mechanical barrier, in particular the apoptosis of IECs and the destruction of TJs, are widely reported in IBD (Coskun, 2014). B. bifidum has been reported to enhance the TJs through a Toll-like receptor-2 (TLR-2) and p38 kinase-dependent pathway (Al-Sadi et al., 2021a). However, Bifidobacteria, especially B. bifidum, exhibited a significant decrease in IBD patients (Duranti et al., 2016). Furthermore, the abundance of SCFA-derived beneficial bacteria reduced in IBD patients (Sokol et al., 2008). SCFAs have been demonstrated to be a key issue in the restore of the intestinal barrier through regulation of TJ proteins and protection of IECs (Wang et al., 2012). SCFAs could upregulate the expression of TJ protein including claudin-1 and Zonula Occludens-1 (ZO-1), and promote the redistribution of occludin (Wang et al., 2012). Meanwhile, butyrate, an important type of SCFAs acting as an energy source, contributes to the proliferation of IECs and reducing their apoptosis (Tremaroli and Bäckhed, 2012). In addition, succinate produced by gut microbiota can promote the specification of tuft cells, which further inhibits the chronic intestinal inflammation in mice (Banerjee et al., 2020). Tuft cells, a rare type of chemosensory epithelial cell in the gut and other mucosal tissues (Strine and Wilen, 2022), play a role in the repair of intestinal epithelium during chronic colitis (Yi et al., 2019). However, several studies reported that tuft cells were significantly reduced in the intestines of UC and CD patients (Banerjee et al., 2020; Kjærgaard et al., 2021).

Contrarily, the increases in harmful and pathogenic microbiota may lead to intestinal mechanical barrier dysfunction via the following pathways: 1) The adherent-invasive E. coli and Fusobacterium nucleatum could attach to IECs and invade mucosal tissue, and further result in excessive intestinal inflammation and intestinal barrier impairment (Darfeuille-Michaud et al., 2004; Brennan and Garrett, 2019; Liu et al., 2020). 2) Pathogen-associated molecular patterns (PAMPs), such as the lipopolysaccharide (LPS) of E. coli (Heimesaat et al., 2007) and the flagellin of Salmonella (Salazar-Gonzalez and McSorley, 2005), are highly conserved structures of microbes (Lim and Staudt, 2013). PAMPs-induced excessive activation of TLRs can result in an increased intestinal barrier permeability (Guo et al., 2013). 3) The harmful microbiota can inhibit the localization and expression of TJ proteins, which further lead to the deterioration of TJs and activation of pro-inflammatory signaling (Jergens et al., 2021). Subsequently, the increased pro-inflammatory cytokines trigger apoptosis of IECs by activating intracellular apoptotic signaling pathways, leading to disruption of the intestinal epithelial integrity and intestinal barrier dysfunction (Iwamoto et al., 1996; Di Sabatino et al., 2003). For example, Salmonella typhimurium induces a strong inflammatory response and disrupts epithelial TJs through its outer proteins such as Salmonella outer protein (Sop) B, SopE, and SopE2 (Jepson et al., 1995; Khan, 2014). 4) The harmful bacteria can activate the apoptosis pathways of IECs, which disrupts the integrity of intestinal barrier. For instance, C.difficile can secrete exotoxin A, which causes colonic epithelial cells to turn round, detach from the basement membrane, and undergo apoptosis (Mahida et al., 1998).

### 2.4 Gut microbiota alteration and immune barrier impairment

The intestinal immune barrier is composed of a vast array of immune cells located within the gut or dispersed across the lamina propria and intestinal epithelium (Perez-Lopez et al., 2016). In IBD patients, pathogens, such as Salmonella and Shigella, or opportunistic pathogens, such as adherent-invasive Escherichia coli (AIEC), pass through a compromised intestinal epithelial barrier, penetrate into the lamina propria (Tawfik et al., 2014), and further activate pattern recognition receptors (PRRs) of immune cells.

PAMPs primarily trigger macrophages and DCs through PRRs, including TLRs and NOD-like receptors (NLRs), and subsequently initiate the pro-inflammatory responses (Walsh et al., 2013). In detail, in the case of gut microbiota dysbiosis, certain pathogens or their products, such as LPS, can excessively activate TLRs on immune cells, especially for TLR4 (Stephens and von der Weid, 2020). The activation of TLRs initiates the downstream signaling pathways, including the MyD88-dependent and TRIF-dependent pathways. The MyD88-dependent pathway activated NF-κB, which upregulates the expression of pro-inflammatory cytokines such as TNF-α, IL-1β, and IL-6, and ultimately results in the amplifying of the inflammatory response. The TRIF-dependent pathway induces the production of type I interferons through interferon regulatory factor 3 (IRF3), which plays a significant role in promoting inflammatory responses (Lawrence, 2009; Lu et al., 2018; Liu et al., 2020).

As pivotal elements of the adaptive immune system, T cells differentiated from naive CD4+ T lymphocytes, such as T helper cells 17 (Th17) and regulatory T cells (Treg), are essential in the progression of IBD (Geremia et al., 2014). Th17 cells aggravate the intestinal inflammation in IBD by secreting IL-17 and IL-22, which are key pro-inflammatory cytokines for activating innate immune cells, promoting neutrophil recruitment, disrupting the intestinal epithelial barrier, and aggravating intestinal inflammation (Rutz et al., 2013; Wu et al., 2016). Gut microbiota contributes to the differentiation and activation of intestinal mucosal immunocytes. A rodent study has demonstrated that transplantation of disorganized gut flora from IBD patients to the germ-free mice resulted in an increase in Th17 cells (Britton et al., 2019). PAMPs from harmful bacteria, such as LPS and flagellin, activate DCs and macrophages, secreting IL-6 and IL-23 (Sica and Mantovani, 2012; Xu et al., 2022), which further promote the differentiation of CD4+ T-cell into Th17 cells via activation of the STAT3 pathway (Yan et al., 2020).

**Figure 2:**
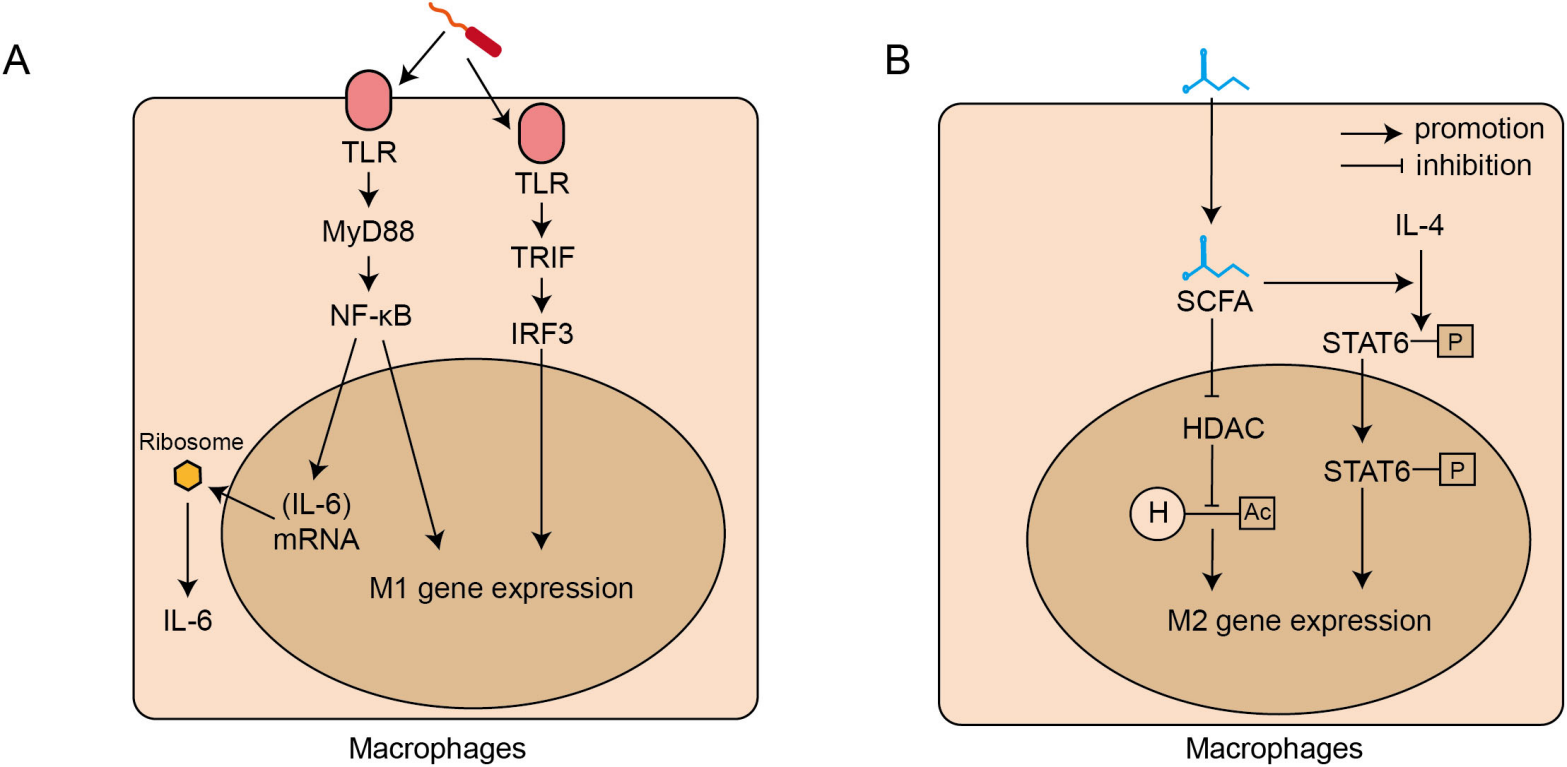
Signaling pathways for intestinal microbiota regulating the polarization and activation of intestinal macrophages. **(A)**. Harmful bacteria-induced M1 polarization and IL-6 secretion: through TLR-MyD88-NF-κb and TLR-TRIF-IF3 signalling pathways. **(B)**. Beneficial bacteria induced M2 polarization: through short chain fat acids (SCFAs) which inhibit HDAC activity and activate the STAT6 signalling pathway. TLRs, toll-like receptors; MyD88, Myeloid Differentiation Primary Response 88; NF-κB, Nuclear Factor kappa-light-chain-enhancer of Activated B cells; TRIF, TIR-domain-containing adapter-inducing interferon-β; IRF3, Interferon Regulatory Factor; M1, macrophage 1 phenotype macrophages; SCFAs, short-chain fatty acids; STAT, Signal Transducer and Activator of Transcription; HDAC, Histone Deacetylase; H, Histone; Ac, Acetyl group; M2, macrophage 2 phenotype macrophages.

Gut microbiota regulates the polarization of monocytes toward different phenotypes of macrophages. On one hand, the harmful microbiota, including the pathogenic ones, promote the polarization of monocytes toward M1 phenotype macrophages through above-mentioned MyD88/NF-κB and TRIF/IRF3 pathway (**Figure 2A**) (Baker et al., 2011). On the other hand, the beneficial bacteria, such as Lactobacillus and Bifidobacterium, play a crucial anti-inflammatory role by maintaining immune homeostasis in IBD, which is mainly mediated via the regulation of SCFAs (Roy and Dhaneshwar, 2023). SCFAs, acting as natural histone deacetylase (HDAC) inhibitors, increase histone acetylation levels, and further induce the polarization of monocytes to M2 phenotype macrophages (Chang et al., 2014). Meanwhile, Butyrate facilitates the IL-4 induced phosphorylation of STAT6, and subsequently increases the mRNA expression of M2-associated genes, including Arg1, Fizz1, and Ym1 (Ji et al., 2016), resulting in the polarization of M0 macrophages toward the M2 phenotype. (**Figure 2B**). M2 phenotype macrophages secrete anti-inflammatory cytokines including TGF-β, which contribute to maintaining an immunosuppressive state (Cutolo et al., 2022). Additionally, TGF-β facilitates the differentiation of naive CD4+Tcells to Tregs, which further amplifies anti-inflammatory responses and promotes homeostasis (Hadis et al., 2011).

In addition to the M2 type macrophage, activated DCs produce immune-suppressive cytokines, including TGF-β and IL-10, when beneficial bacteria interacts with PRRs of DCs (Ghavami et al., 2020).The maturation and activation of DCs in the mesenteric lymph nodes enhance their antigen-presenting properties, which supports immune tolerance and promote differentiation of naive T cells into Tregs (Coombes et al., 2007). For example, Faecalibacterium prausnitzii (F. prausnitzii) interacts with TLR2/6 receptors of DCs, activate the MAPK-JNK signaling pathway and enhances the expression of anti-inflammatory factors, IL-10 and IL-27, which promotes the generation/differentiation of Tregs (Alameddine et al., 2019; Amoroso et al., 2020). In IBD patients, a decrease in the abundance of F. prausnitzii leads to a notable reduction in intestinal Tregs, which subsequently diminishes the inhibition on the colonic Th17 cells (Ohnmacht et al., 2015). IL-25, another anti-inflammatory factor mainly secreted by Tuft cells, could inhibit the activation of CD4 + T cell and inhibit their differentiation into T helper 1 (Th1)/Th17 cells via an IL-10-dependent pathway in IBD (Su et al., 2013). However, both tuft cells and IL-25 are significantly reduced in IBD patients.

## 3 Microbiota-targeted treatment options for inflammatory bowel disease

The interaction between gut microbiota dysbiosis and the impaired intestinal barrier plays the crucial roles in the pathophysiology of IBD.As the microbiota-targeted treatment for the improvement or restoration of the intestinal microecology in IBD, antibiotics, probiotics and FMT exhibit their advantages, deficiencies and synergistic effects (see **Table 1**) when used alone or in combination.

**Table 1:** Comparison of microbiota-targeted therapies in Inflammatory bowel disease: antibiotics, probiotics and fecal microbiota transplantation.

Microbiota-targeted therapies	Antibiotics	Probiotics	FMT
Commonly used drugs, strains, or donors.	Metronidazole, Ciprofloxacin, Rifaximin	Traditional probiotics: Lactobacillus, Bifidobacterium New probiotics: F. prausnitzii, Roseburia spp, Akk	Fecal microbiota from healthy donors
Therapeutic mechanism	①Reducing the adherence, invasion, translocation and systemic diffusion of pathogens. ②Inhibiting overgrowth of harmful bacteria, and alters the diversity and construction of gut microbiota. ③Reducing the pro-inflammatory cytokines.	①Inhibition of potential pathogens. ②Production of beneficial metabolites or enzymes. ③Immune modulation.	①Restoring the gut microbiota. ②Restoration of healthy gut microbiota and gut barrier function. ③Regulation of immune response. ④Inhibition of intestinal inflammation.
Impact on the gut microbiota	①Traditional antibiotics, such as Metronidazole, reduce the diversity of the gut microbiota. ②Selective intestinal antibioticof Rifaximin can produce a favorable gut microbiota perturbation without affecting the overall composition of the gut microbiota.	Increasing the abundance of probiotics.	①Restoring gut microecological homeostasis. ②Increasing the abundance of beneficial gut microbiota and reducing the detrimental ones. ③May lead to a sustained microbiota restoration.
Clinical efficacies in existing studies	Clinical remission	Alleviating GI symptoms	Clinical remission, steroid-free remission, or endoscopic remission
Indication/ Applicable situations	Postoperative period, pathogen infections, and septic complications, including wound infections, perianal fistulas, and intra-abdominal abscesses.	especially in UC, but limited evidence in CD	UC (especially for refractory ones), but limited evidence in CD; IBD with refractory C. difficile infection.
Recommendations or evaluations in guidelines and consensus	WGO Global Guidelines: Metronidazole and ciprofloxacin are the most commonly used antibiotics in CD. They are used in the treatment of CD complications, including perianal lesions, fistulas, inflammatory masses, and bacterial overgrowth in cases of strictures	①British Society of Gastroenterology consensus guidelines suggest that probiotic therapy may offer some benefit to UC patients, but it should not be used routinely. In contrast to UC, there is currently no sufficient evidence to support the benefit of these probiotic therapies for Crohn's disease. ②AGA Clinical Practice Guidelines: The use of probiotics is only recommended in the context of a clinical trial in adults and children with UC or CD. ③ECCO guidelines: Treatment of probiotics in IBD patients receiving anti-TNF therapies is probably safe, but still remaining a safety concern for probiotics with beta-haemolytic activity	First international Rome consensus conference on gut microbiota and FMT in IBD: ①Previously performed RCTs are small and methodologically heterogeneous; thus, definitive conclusions cannot be drawn at the present time. ②FMT is recommended as a treatment option for both mild and severe recurrent or refractory C. difficile infection in patients with IBD. ③FMT may be effective in the induction of remission in mild to moderate UC; however, there is insufficient evidence to recommend FMT as a treatment for UC in routine clinical practice and its use should generally be limited to the research setting. ④Insufficient evidence to recommend FMT as a treatment for CD in clinical practice. To date, its use should be limited to the research setting.
Risks,limitations and unresolved issues	① The long-term antibiotic treatment is considered as a possible trigger for the progression of IBD. ② Risk of disturbing immune homeostasis and aggravating intestinal inflammation. ③ Risk of resistance, gastrointestinal disturbances.	①Risk of bacterial translocation across the impaired intestinal barrier, leading to systemic infections. ②Risk of transferring antibiotic resistance genes to pathogenic bacteria. ③ Limitations of undefined and unsustained efficacy, especially in CD. Further clinical validation is needed to establish their therapeutic potential	①Risk of infections, donor variability, uncertain long-term effects. ②The undefined optimal donor selection (multiple donors/single donor, Frozen/fresh) and administration route (upper/lower GI route). ③More large-scale, multi-center randomized controlled trials are needed to further confirm the efficacy of FMT in IBD.

FMT, fecal microbiota transplantation; F. prausnitzii, Faecalibacterium prausnitzii; Akk, Akkermansia muciniphila; C. difficile, Clostridioides difficile; IBD, inflammatory bowel disease; UC, ulcerative colitis; CD, Crohn's disease; WGO, World Gastroenterology Organization; AGA, American Gastroenterological Association; ECCO, European Crohn’s and Colitis Organization; GI, gastrointesinal.

### 3.1 Antibiotic therapy in IBD

The antibiotics are applied as a reliable therapy in IBD patients (Khan et al., 2011), although they are described as “deep modulators of gut microbiota between good and evil” (Ianiro et al., 2016). The antibiotics exhibit benefits through various potential mechanisms. Firstly, antibiotics can reduce the adherence, invasion, translocation and systemic diffusion of pathogens (Ledder and Turner, 2018). Secondly, antibiotic treatment alters the diversity and construction of gut microbiota. It inhibits the overgrowth of harmful bacteria, and indirectly provide a favorable environment for the survival of beneficial bacteria. Thirdly, antibiotics can reduce the pro-inflammatory cytokines which might due to both antibacterial and immunoregulatory properties (Garrido-Mesa et al., 2011). The common antibiotics used in IBD include systemic antibiotics such as ciprofloxacin and metronidazole, as well as the intestinal-selective antibiotic rifaximin (Biancone et al., 2017). For instance, metronidazole, one of the narrow spectrum antibiotics, has been applied in CD patients for eliminating the bacterial overgrowth and blocking the bacterially mediated antigenic triggers (Lichtenstein et al., 2018). The typical prescription involves 500mg of metronidazole administrated orally or intravenously every 8 hours for a duration of 7–10 days. Rifaximin is another key antibiotic used in the treatment of IBD (Biancone et al., 2017). In addition to its direct bactericidal activity, Rifaximin has also been shown to inhibit intestinal bacterial translocation, reduce the adhesion and internalization of pathogenic bacteria (Fiorucci et al., 2002; Brown et al., 2010), and inhibit the expression of pro-inflammatory factors (Cheng et al., 2010). A clinical trial has shown that Rifaximin can induce and maintain remission in patients with moderate active Crohn’s disease (Prantera et al., 2012). Interestingly, Maccaferri S et al. showed that Rifaximin did not affect the overall composition of the gut microbiota, but it did lead to an increase in the concentration of Bifidobacterium, Atopobium, and F. prausnitzii (Maccaferri et al., 2010). This finding differentiates Rifaximin from other common antibiotics. By producing a favorable gut microbiota perturbation, Rifaximin holds the potential to open new horizons for its use in a specific group of CD patients (Lopetuso et al., 2018). Additionally, as a selective intestinal antibiotic, Rifaximin acts locally in the gut, resulting in fewer side effects and helping to avoid systemic resistance issues, making it particularly advantageous in the treatment of IBD (Biancone et al., 2017). Although widely administrated in IBD, antibiotics cannot replace first-line anti-inflammatory agents such as amino salicylates, corticosteroids, or immunosuppressants in the treatment of IBD patients (Lichtenstein et al., 2018; Rubin et al., 2019). The more widely accepted view is that antibiotic treatments might benefits IBD patients in certain circumstances such as postoperative period, pathogen infections, and septic complications including wound infections, perianal fistulas and intra-abdominal abscesses (Jha et al., 2024).

Most of the evidence suggests that short-term microbiota-targeted therapeutic agents trigger rapid alterations in the diversity and construction of microbiota, whereas the altered gut microbiota restores to its pre-treatment state once treatment is completed (Berg et al., 2015).However, the long-term antibiotic treatment is considered as a possible trigger for the progression of IBD (Theochari et al., 2018). It has been demonstrated that patients who previously received treatments of three or more antibiotics presented a 55% increased risk of developing IBD compared to those who never used antibiotics (Sokol, 2020). Based on early data from 24,000 IBD patients, a national population-based study in Sweden showed a significant increased risk of IBD which is associated with high cumulative exposure to systemic antibiotic therapy, particularly applications of broad-spectrum antibiotics (Nguyen et al., 2020). Antibiotic-related risk of IBD further increases in individuals aged 40 years or more and during cumulative antibiotic exposure or 1–2 years after antibiotic exposure (Faye et al., 2023).

Antibiotic treatments, especially long-term and/or broad-spectrum antibiotic treatments reduce the abundance of various gut microbiota, including beneficial bacteria (Jernberg et al., 2007; Kim et al., 2017), which subsequently disturbs immune homeostasis and aggravates intestinal inflammation (Yoon and Yoon, 2018). The antibiotics significantly alter the diversity of the gut microbiota, hinder the long-term reconstruction of the gut microbiota, and promote the differentiation of CD4+ T cells toward a Th17 pro-inflammatory phenotype (Burrello et al., 2018). Meanwhile, antibiotics induced disruption of the gut microbiota results in an overreaction of intestinal macrophages which produce excess pro-inflammatory cytokines (Scott et al., 2018); Further, re-exposure of antibiotic-treated mice to common microbiota induces an increased susceptibility of macrophage-dependent inflammatory Th1 cells and persistent dysbiosis in the colon (Scott et al., 2018). Accordingly, antibiotics serve as a double-edged tool in the treatment of IBD, further researches with high-level evidences are warranted for more reasonable antibiotic therapy in IBD.

### 3.2 Probiotic treatment in IBD

Probiotics are live microorganisms that provide a health benefit to the host when taken in sufficient quantities. According to the previous reports, when Lactobacillus and Bifidobacterium were delivered in food at a level of 1 × 10^9 colony-forming units (CFU) per serving, they were accepted and recognized as probiotics by Health Canada and the Italian Ministry of Health (Hill et al., 2014). Alternatively, the expert panel of the International Scientific Association for Probiotics and Prebiotics indicated that certain potential mechanisms of probiotics, such as the inhibition of potential pathogens or the production of beneficial metabolites or enzymes, are widespread among various strains, while other effects, such as immune modulation, are only present in specific strains (Hill et al., 2014). Probiotics, as a potential adjunctive therapy, have garnered increasing attention in the treatment of IBD, particularly in alleviating symptoms, reducing intestinal inflammation, and restoring gut microbial homeostasis (Bjarnason et al., 2019; Jakubczyk et al., 2020; Martín et al., 2023). Additionally, the application of probiotics carries potential safety concerns. probiotics are considered to have the potential for bacterial translocation across the intestinal barrier. When intestinal barrier is severely damaged in IBD, long-term colonized probiotics may enter the bloodstream, leading to systemic infections (Rouanet et al., 2020). European Crohn’s and Colitis Organization (ECCO) guidelines demonstrates that treatment of probiotics in IBD patients receiving anti-TNF therapies is probably safe, but still remaining a safety concern for probiotics with beta-haemolytic activity (Kucharzik et al., 2021). Furthermore, probiotics may carry antibiotic resistance genes, which can be transferred to pathogenic bacteria through horizontal gene transfer, thereby complicating treatment (Merenstein et al., 2023).

Lactobacillus (e.g. strains of acidophilus, casei, fermentum, gasseri, johnsonii, paracasei, plantarum, rhamnosus and salivarius) is widely recognized as one of the representative strains of probiotics in the adjuvant treatment of IBD, which plays a vital role in alleviating intestinal injury, strengthening intestinal barrier function, and regulating immune responses (Li et al., 2023). Lactobacillus can secrete antimicrobial substances, such as bacteriocins and hydrogen peroxide, which inhibit the proliferation of pathogens (Dempsey and Corr, 2022) and other harmful bacteria, such as Campylobacter jejuni, Salmonella Enteritidis, and E.coli (Carter et al., 2017; Chingwaru and Vidmar, 2017; Saint-Cyr et al., 2017). A specific strain of Lactobacillus acidophilus has been reported to strengthen the TJs of intestinal mechanical barrier through TLR-2 (Al-Sadi et al., 2021b). Lactobacillus rhamnosus has been shown to promote the expression of Muc2 and Muc3 in goblet cells in murine intestinal inflammation models, thereby enhancing mucus production to strengthen the intestinal chemical barrier (Martín et al., 2019). Additionally, Lactobacillus casei has been shown to strengthen the intestinal immune barrier, mediated via the activation of Treg cells, upregulation of IL-10, and reduction of TNF-α and IL-12, thus reflecting an anti-inflammatory effect in IBD (Liu et al., 2021).

Bifidobacteriaceae (e.g. strains of adolescentis, animalis, bifidum, breve and longum) has been well recognized as a beneficial microbiota in IBD patients. Probiotic therapy with Bifidobacteria alleviates symptoms in IBD patients (O’Callaghan and van Sinderen, 2016). Several studies reported that Bifidobacteria play an important role in maintaining the integrity of intestinal epithelial barrier (Khailova et al., 2009; Bergmann et al., 2013; Hsieh et al., 2015; Martín et al., 2016).A recent rodent study on trinitrobenzene sulfonic acid induced colitis further indicated that Bifidobacteria alleviate the colitis by upregulating the expression of indoleamine 2,3-dioxygenase, which further increases the Treg cells (Zhao et al., 2018). Bifidobacteria can produce health-promotors including vitamins and SCFAs, which strengthen the intestinal barrier and modulate the host’s immune response (Al-Sadi et al., 2021a). One study in Caco-2 monolayers showed that B. bifidum contributed to a marked, sustained enhancement on the intestinal TJs of mechanical barrier and protected against intestinal inflammation through targeting TLR-2 and via an NF-κB-independent pathway (Al-Sadi et al., 2021a). Meanwhile, it was indicated that B. bifidum regulated the host’s immune response and reduces the expression of TNF-α, exerting an anti-inflammatory effect (Chae et al., 2018).In addition to traditional probiotics including Lactobacillus and Bifidobacteria, new generation of probiotics, such as F. prausnitzii, Roseburia spp. and Akk, has being developed for the treatment of IBD (Al-Fakhrany and Elekhnawy, 2024). F. prausnitzii belongs to family Oscillospiraceae, works as the key bacteria that produce butyrate, an important SCFAs (Louis and Flint, 2017).Several researches suggest that butyrate plays a crucial role in maintaining gut homeostasis, including defense against pathogen colonization, restoration of the TJs, and modulation of the immunoreaction (Plöger et al., 2012; Miquel et al., 2013). Meanwhile, F. prausnitzii is also well known for its immunomodulatory properties, exerting an anti-inflammatory effect by upregulating the expression of Dact3, a gene linked to the WntJNK pathway (den Besten et al., 2013; Lenoir et al., 2020). Meanwhile, F. prausnitzii might promote the Tregs differentiation/expansion via an IL-10-dependent pathway, which mediate the tolerance to inflammation signals (Sarrabayrouse et al., 2014). It has been widely shown that the abundance of intestinal F. prausnitzii is significantly reduced in both CD and UC patients compared to healthy controls; meanwhile, IBD patients in active stage showed a significant lower abundance of F. prausnitzii than those in remission stage (Zhao et al., 2021). FMT therapy can partially reverse the abundance of F. prausnitzii, which also aids in modulating the intestinal Th17/Treg balance and alleviates intestinal inflammation (Huang et al., 2022b). Roseburia is known for its positive effects on IBD, particularly as a butyrate-derived bacterium (Vacca et al., 2020). In addition, Roseburia intestinalis contributes to restoration of the gut microbiota through upregulated expression of IL-22 and restoration of the intestinal barrier integrity through upregulation of the Occludin, one of the important TJ proteins. Accordingly, the abundance of Roseburia is negatively correlated with the activity of UC (Machiels et al., 2014). Akk is a new probiotic with great potential in the treatment of IBD through various mechanisms, including modulation of gut microbial homeostasis, immune response, inhibition of pathogen colonization, and enhancement of intestinal barrier function (Zheng et al., 2022). Studies have shown that both live Akk and inactivated Akk can alleviate DSS-induced colitis in mice (Bian et al., 2019; Qian et al., 2022; Zheng et al., 2023). Inactivated Akk acts through several components including outer membrane protein Amuc_1100, enzyme Amuc_2109 and extracellular vesicles (AmEVs). AmEVs has been reported to regulate intestinal barrier permeability by regulating the expression of TJ proteins (Chelakkot et al., 2018). Akk can regulate the differentiation of Tregs, increase production of SCFA, downregulate the expression of pro-inflammatory cytokines (including TNF-α and IFN-γ) in the colon of mice, and promote the restoration of the gut microbiota (Zhai et al., 2019). Emerging researches also highlights other promising probiotics like Christensenella minuta, Anaerostipes spp., Oscillospira spp., and Saccharomyces boulardii in reducing IBD risk and improving gut barrier function, supported by their anti-inflammatory butyrate production and microbiota-modulating effects. However, further clinical validation is needed to establish their therapeutic potential (Jan et al., 2024).

Overall, existing studies have demonstrated the therapeutic potential of probiotics in the treatment of IBD. However, probiotic therapy may only serve as a complementary treatment for IBD. According to the American Gastroenterological Association (AGA) Clinical Practice Guidelines, the use of probiotics is only recommended in the context of a clinical trial in adults and children with UC or CD (Su et al., 2020). The British Society of Gastroenterology consensus guidelines suggest that probiotic therapy may offer some benefit to UC patients, but it should not be used routinely. In contrast to UC, there is currently no sufficient evidence to support the benefit of these probiotic therapies for Crohn’s disease (Lamb et al., 2019). High-quality, well-designed, multi-center and large sample studies are needed to provide high-level evidence for the application of probiotics in the future.

### 3.3 FMT treatment in IBD

FMT, a new therapeutic option for IBD, has attracted increasing attention for its potential benefits in IBD patients by restoring gut microecological homeostasis (Imdad et al., 2023). FMT refers to the transfer of healthy donor feces into the recipient’s GI tract, in order to restoring the gut microbiota to treat intestinal and extra-intestinal diseases. FMT is recommended in the guidelines for treating recurrent C. difficile infection (CDI) (Cammarota et al., 2017; Kucharzik et al., 2021), and is also considered to reveal therapeutic potential in the treatment of other diseases, such as IBD, irritable bowel syndrome (IBS), and functional constipation (Cammarota et al., 2017). A large number of evidences establish the beneficial efficacies of FMT in patients with IBD by restoring the disordered gut microbiota, increasing the abundance of beneficial gut microbiota and reducing the detrimental ones (Pai et al., 2021; Huang et al., 2022a; Kedia et al., 2022).

Recent randomized controlled trials (RCTs) have indicated that FMT exhibited a therapeutic property for the remission of UC (see **Table 2**). Most of these RCTs (5/6) demonstrated that FMT is more effective in inducing clinical and/or endoscopic remission in UC patients compared to the control group. Brezina et al (Březina et al., 2021). and Fang et al (Fang et al., 2021). indicated that FMT therapy resulted in noninferior or higher clinical remission rate compared to the traditional treatment of 5-aminosalicylic acid (5-ASA) and/or steroid. Compared to those treated with water enema (Sham-FMT) in placebo group, Moayyedi et al (Moayyedi et al., 2015). showed that 6 times of FMT treatments from single donor (FMT-SDN) resulted in a significant higher clinical remission rate (24% *vs*. 7%, *P*=0.03). Meanwhile, Costello et al (Costello et al., 2019). and Paramsothy et al (Paramsothy et al., 2017). revealed significant higher rate of Steroid-free clinical and/or endoscopic remission though FMT from multiple donors (FMT-MDN) in comparison with FMT with autologous feces (FMT-A) or Sham-FMT (isotonic saline enema). However, Rossen et al (Rossen et al., 2015). showed that 2 times of FMT-SDN (n=15) showed no significant difference on clinical remission rate in UC patients in comparison with that of FMT-A (n=8), which might be attributed to the limited sample size and FMT administration frequency.

**Table 2:** Randomized controlled trials (RCTs) of Fecal microbiota transplantation (FMT) treatment for ulcerative colitis (UC) patients.

Research	Patient criteria	Sample size	Treatment of FMT group			Treatment of control group	Clinical remission		Primary endpoint	Statistics	
		FMT/Control (n/n)	Donor	Stool storage	FMT times		FMT	Control		Noninferiority trial	Chi-square test
Březina 2021 (Březina et al., 2021)	①age<70 ②endoscopically active left-sided UC ③Mayo score 4-10 ④endoscopy subscore ≥2	21/22	single	Frozen −80°C	10	5-ASA	57%	36%	Clinical remission rate at week 12	10% (95% CI: -7.6% to 48.9%)	–
Fang 2021 (Fang et al., 2021)	①age 18-75 ②Mayo score 4-12	10/10	single	fresh	1	5-ASA Steroid	90%	50%	Clinical remission at week 8	–	*P* = 0.019
Moayyedi 2015 (Moayyedi et al., 2015)	①age ≥18 ②active UC ③Mayo Clinic score ≥4 ④endoscopic Mayo Clinic score ≥1	38/37	single	Frozen −20°C	6	water	24%	5%	Clinical remission at week 7	–	*P* = 0.030
Costello 2019 (Costello et al., 2019)	①age ≥18 ②active UC ③Mayo score 3-10 ④endoscopic subscore of ≥2	38/35	Multiple (3-4 donors)	anaerobic conditions, Frozen −80°C	2	autologous feces	32%	9%	Steroid-free remission at week 8	–	*P* = 0.030
Paramsothy 2017 (Paramsothy et al., 2017)	①age 18-75 ②endoscopically and clinically active ulcerative colitis ③Mayo score 4-10 ④endoscopy subscore ≥ 1 ⑤assessment subscore ≤ 2	41/40	Multiple (3-7 donors)	Frozen −80°C	40	isotonic saline	27%	8%	Steroid-free clinical remission with endoscopic remission or response at week 8	–	*P* = 0.021
Rossen 2015 (Rossen et al., 2015)	①UC according to the Lennard-Jones criteria ②SCCAI ≥4 and ≤11 ③stable medication	17/20	single	fresh	2	autologous feces	26.10%	32%	Clinical and endoscopic remission at week 12	–	*P* = 1.000

A search was conducted on PubMed using the query "((FMT[Title/Abstract]) AND ((Ulcerative Colitis [Title])" resulting in the identification of 23 randomized controlled trials (RCTs) which performed FMTs via the lower gastrointestinal tract route for treating UC. After reviewing titles and abstracts, 17 studies were excluded. The remaining 6 research exhibited the efficacy of FMT on UC.

The variations in donor selection and treatment intensity may contribute to the different clinical outcomes. The higher bacterial species richness in donors are associated with successful transplantation in FMT treatment in UC patients (Vermeire et al., 2016). In the above-mentioned RCTs in **Table 2**, 3 of them used FMT-SDN, whereas Costello et al. and Paramsothy et al. applied FMT-MDN. Compared to patients received FMT-SDN, patients received FMT-MDN achieved steroid-free remission at the primary endpoint. A meta-analysis that included 14 studies indicated that FMT-MDN showed superior treatment response in IBD compared to placebo or FMT-SDN (Levast et al., 2023). Another study also revealed that pooling stools from multiple donors to increase microbial diversity could enhance remission rates in UC patients (Kazerouni and Wein, 2017). FMT-MDN in IBD patients offers broader microbial diversity that aids in restoring the gut microbial flora homeostasis and improving treatment efficacy. However, FMT-MDN results in complicated procedures and increased resource consumption, while FMT-SDN is more stable, with simple procedure and easy storage. In addition, a systematic review and meta-analysis showed that FMT-MDN and FMT-SDN were similar in therapeutic safety; meanwhile, FMT-MDN treatment demonstrated a better efficacy in UC patients, thus FNT-MDN indicated a greater benefit-risk ratio than FMT-SDN (Laperrousaz et al., 2024).

The Rome consensus indicates that donor feces, whether fresh or frozen, can be used in FMT. The frozen FMT samples are preferred over fresh preparations, primarily due to safety concerns (Lopetuso et al., 2023). However, it is still controversial whether fresh or frozen feces provides superior efficacy in FMT for IBD patients. A systematic review, which included a total of 14 trials, indicated no significant difference in clinical remission rate between FMT using fresh stool (40.9%) and frozen stool (32.2%) (Tan et al., 2022). However, Cheng et al. showed that fresh stool FMT yields a higher clinical remission rate than frozen stool FMT (73% *vs*. 43%, *P* < 0.05) (Cheng et al., 2021). Agarwal et al. indicated that larger volumes of fresh stool may be more effective than smaller amounts of frozen stool for treating recurrent or refractory C. difficile infection (rCDI) (Agarwal et al., 2021).

The acceptable and effective routes of administration are also an important issue in FMT (Gulati et al., 2020). The delivery of donor’s fecal microbiota to recipient’s GI tract can be carried out through the upper, middle, and lower GI routes (Cui et al., 2015; Zhang et al., 2020; Crothers et al., 2021). A meta-analysis of 14 trials showed that the clinical remission rate for FMT through the lower GI tract was similar to that through the upper GI tract (38.2% [151/395] *vs*. 31.2% [15/48]), *P*>0.05), indicating no significant difference in remission rate due to the different routes of administration (Tan et al., 2022). However, another meta-analysis containing 7 studies on treatment of UC revealed FMT-MDN using frozen donor stool delivered through the lower GI tract was more effective than placebo; whereas no significant difference on efficacy was recorded between FMT transplanted via the upper GI tract and placebo (Tang et al., 2020). Although it still remains unclear which route acts as optimal one regarding the efficacy of FMT, physicians typically take into account additional key factors such as patient compliance, cost-effectiveness, comfort of administration, invasiveness, risk of aspiration and infection, the number of drugs to be administered, and relapse rate when selecting the route of FMT administration (Gulati et al., 2020). For example, the upper and middle GI route, including infusion through nasogastric, nasojejunal, gastrostomy or jejunostomy tube, might result in psychological difficulties in IBD patients (Peng et al., 2016; Chen et al., 2025). However, encapsulated oral FMT for a long-term therapy in UC is considered to be safe and well accepted (Crothers et al., 2021). The lower GI routes encompass enemas, colonoscopy, colostomy, and transendoscopic enteral tubing (Peng et al., 2016). Notably, FMT through transendoscopic enteral tubing (TET) for IBD has been reported to have a high acceptance (Lin et al., 2024). Alternatively, there is growing evidence that colonic administration, rather than nasoduodenal administration, may be safer and more effective (Kelly and Ananthakrishnan, 2019).

Compared to the higher acceptance of FMT in the treatment of UC, the efficacy of FMT in CD needs to be confirmed by further research. A small-sample study indicated the more than 40%higher steroid-free clinical remission rate at week10 in FMT group compared to that of control group (Sham-FMT), but the difference did not reach statistical significance, which might be due to the limited sample size (Sokol et al., 2020). However, another research showed that multiple FMTs (repeated FMTs every 3 months) can induce and maintain clinical remission in CD with complication of inflammatory mass (He et al., 2017).

By introducing a diverse and balanced array of microbial species, FMT has been shown to significantly increase the abundance of beneficial gut microbiota (Zhi-Ning et al., 2024), which plays a key role in restoring the gut microecological homeostasis in IBD patients. FMT induced remission in IBD patients is highly associated with the increased abundance of beneficial gut microbiota, such as families Oscillospiraceae, Clostridiaceae and Lachnospiraceae of the phylum Bacillota, as well as Coriobacteriaceae and Bifidobacteriaceae of the phylum Actinobacteria (Březina et al., 2021).

In comparison with the probiotics which are typically limited in establishing a long-term colonization in the gut, FMT can result in a sustained microbiota restoration, with microbial communities from the donor to the recipient’s GI tract persisting for extended periods, potentially contributing to a long-term therapeutic effect (Weingarden et al., 2015). FMT is potential to restore the diversity of the gut microbiota, which is often compromised in IBD patients, by reintroducing a wide array of microbial species with the corresponding metabolites and functions (Hourigan et al., 2015). One study indicated that certain probiotic strains might become undetectable within two weeks after cessation of supplementation (Maldonado-Gómez et al., 2016). However, FMT has been shown to facilitate long-term engraftment of microbial species in the recipients’ gut.

### 3.4 Combined therapy of antibiotic and FMT in IBD patients

#### 3.4.1 Antibiotic pretreatment of FMT

During the preparation process for FMT recipients, some researchers employ broad-spectrum antibiotics in conjunction with classic colon cleansing with polyethylene glycol as a pretreatment strategy (Pigneur and Sokol, 2016). This procedure aims to reduce the existing gut microbial load and create a favorable environment for the transplanted microbiota (Suez et al., 2018). In a recent RCT, 2-week of antibiotics including amoxicillin, metronidazole, and doxycycline were administrated in active UC patients, followed by 8-week treatment of oral lyophilised FMT or placebo. It indicated that FMT with antibiotic pretreatment showed a significant higher corticosteroid-free clinical remission rate in comparison with those with placebo pretreatment (Haifer et al., 2022). Furthermore, several studies indicated that a single FMT with the pre-treatment of combined antibiotics resulted in a notable higher clinical remission rate in comparison with multiple FMT without antibiotic pre-treatment in IBD (Moayyedi et al., 2015; Ishikawa et al., 2022; Zhang et al., 2022). The pre-treatment of antibiotic might enhance the efficacy of FMT through reducing the luminal microbial colonies and aiding in microbial restoration (Ishikawa et al., 2017; Kump et al., 2018). The pretreatment of antibiotics facilitates the successful transfer and colonization of donor microbiota which enhance the overall therapeutic effectiveness of FMT (Ji et al., 2017; Smith et al., 2022). Although these findings highlight the importance of antibiotic pretreatment in improving FMT outcomes, further RCTs are warranted to confirm the necessity and procedure of the antibiotic pretreatment of FMT.

#### 3.4.2 FMT reverse antibiotic- induced dysbiosis

FMT contributes to the reconstruction of the gut microbiota which was disturbed by antibiotics. A rodent study showed that both antibiotics and chemotherapy treatments significantly altered the gut microbiota, reflecting as the reduced varieties and abundance of microbial species, which were quickly restored 1 week after FMT (Le Bastard et al., 2018). Further, antibiotic exposures, especially long-term and broad-spectrum ones, can lead to an imbalanced symbiotic bacteria in the colon, further resulting in gut dysbiosis and potentially CDI (Kim and Gluck, 2019). FMT not only improves the intestinal microecology but also restores the intestinal function in patients with CDI (Brandt et al., 2012). FMT treatment on CDI is mainly attributed to its direct and indirect suppression of C. difficile. The restored gut microbiota after FMT treatment directly competes with C. difficile for nutrients (Choi and Cho, 2016), and resists against its colonization (Britton and Young, 2012). Meanwhile, FMT indirectly suppresses C. difficile by reviving the metabolism of secondary bile acids, which subsequently inhibit the germination of C. difficile spores (Heath et al., 2018).

Moreover, the repeated cycles of antibiotic treatments for rCDI continuously aggravates the colonic microbiota dysbiosis, which results in an increased risk of rCDI-associated diarrhea. However, FMT has been identified as an effective and safe treatment for rCDI (Cammarota et al., 2014). In addition to reversing disturbed gut microbiota after antibiotic treatment, FMT may also play a crucial role in restoring the injured intestinal barrier in rCDI (Samarkos et al., 2018).

## 4 Conclusion

Gut microbiota dysbiosis and associated intestinal barrier damage play key roles in the pathophysiology of IBD. The microbiota-targeted treatment options for IBD, including antibiotics, probiotics, and FMT alone and in combination in the treatment of IBD, contribute to the restoration of healthy gut microbiota and gut barrier function, regulation of immune response, and inhibition of intestinal inflammation.

These microbiota-targeted interventions might provide new insights for IBD management. Furthermore, well-designed RCTs and animal experiments involving mechanism of maintaining intestinal micro homeostasis are essential to optimize microbiota-targeted interventions, aiming to provide safer and effective treatment options for IBD.

## 5 Perspective

Dysbiosis of the gut microbiota contributes to the progression of IBD via the alteration of metabolism, impairment of intestinal barrier, and dysregulation of immune responses. However, several items involving the mechanism and therapeutic options targeted to gut microbiota in IBD requires further researches. 1) whether gut dysbiosis acting as a trigger or aggravator for IBD or the secondary alteration of intestinal inflammation remains ambiguous. Existing studies suggest that there is a bidirectional interaction between the gut microbiota and IBD. On one hand, dysbiosis of the gut microbiota may trigger or aggravate IBD by initiating abnormal immune responses and disrupting the intestinal barrier (Brand, 2009; Stange and Schroeder, 2019). On the other hand, the inflammatory state of the intestinal microenvironment in IBD may further alter the structure and function of the gut microbiota, creating a vicious cycle (Stange, 2024). For example, research has found that early-stage IBD patients already exhibit microbiota imbalance, suggesting that microbiota dysbiosis may play a triggering role in the pathogenesis of IBD. However, there is also evidence indicating that inflammation itself can significantly alter the intestinal microbial ecosystem, exacerbating microbiota dysbiosis (Khan et al., 2019). Therefore, conducting longitudinal studies to track the dynamic changes in microbiota structure in high-risk populations and patients is of great significance in clarifying the temporal sequence and causal relationship between dysbiosis and IBD progression, which is crucial for the formulation of precise prevention and treatment strategies in the future. Additionally, the altered gut microbiota varies among individuals and various stages of IBD. Future research is highlighted to explore key microbial species or core indicators of healthy microbiota, and to study the pivotal pathways by which the key microbiota acts on the regulation of intestinal barriers and immune response in IBD. 2) Further studies on pathophysiology of IBD might focus on a multidisciplinary perspective, integrating microbiota studies with genomics, metabolomics, and immunology, in order to establish and optimize the new therapeutic regimens. 3) Multicentral, well-designed RCTs are warranted for the guidelines of antibiotic, probiotic and FMT applications in IBD, involving the reasonable indication, optimal types, dosages, course and combined applications with other treatments. 4) The important role of the gut microbiota in host immune regulation, metabolic function, and intestinal barrier function has made it a crucial biomarker for predicting disease risk, treatment response, and disease progression (Alexandrescu et al., 2024b; Dumitru et al., 2024). Alexandrescu et al. indicated that the notable altered composition of the gut microbiota in active phase of IBD patients may be highly related to the severity of the disease and treatment response (Alexandrescu et al., 2024a). Analyzing the composition of the gut microbiota in conjunction with clinical data might offer a foundation for personalized treatment (Alexandrescu et al., 2024a). A systemic review indicated that the IBD patients who responded to treatment of anti-interleukin or anti-tumor necrosis factor reflecting constantly (both at baseline and throughout the therapy) higher α-diversity and increased relative abundances of certain genera such as Faecalibacterium, Roseburia, or Clostridium (Estevinho et al., 2020). Accordingly, prospective studies are warranted to determine the key species of microbiota and the crucial parameters regarding their composition, function, or metabolites which could be used as the biomarker for diagnosis and prediction of prognosis, risk and treatment response.
